# Fluctuations in Arousal Correlate with Neural Activity in the Human Thalamus

**DOI:** 10.1093/texcom/tgab055

**Published:** 2021-09-01

**Authors:** Tetsuya Iidaka

**Affiliations:** Brain & Mind Research Center, Nagoya University, Nagoya, Japan

**Keywords:** awake, blink, consciousness, fMRI, rest

## Abstract

The neural basis of consciousness has been explored in humans and animals; however, the exact nature of consciousness remains elusive. In this study, we aimed to elucidate which brain regions are relevant to arousal in humans. Simultaneous recordings of brain activity and eye-tracking were conducted in 20 healthy human participants. Brain activity was measured by resting-state functional magnetic resonance imaging with a multiband acquisition protocol. The subjective levels of arousal were investigated based on the degree of eyelid closure that was recorded using a near-infrared eye camera within the scanner. The results showed that the participants were in an aroused state for 79% of the scan time, and the bilateral thalami were significantly associated with the arousal condition. Among the major thalamic subnuclei, the mediodorsal nucleus (MD) showed greater involvement in arousal when compared with other subnuclei. A receiver operating characteristic analysis with leave-one-out crossvalidation conducted using template-based brain activity and arousal-level data from eye-tracking showed that, in most participants, thalamic activity significantly predicted the subjective levels of arousal. These results indicate a significant role of the thalamus, and in particular, the MD, which has rich connectivity with the prefrontal cortices and the limbic system in human consciousness.

## Introduction

Consciousness is unequivocally the most fundamental construct of thought and behavior in human beings; therefore, the exact nature of consciousness has been explored in multiple disciplines. However, the essential characteristics and qualities of consciousness remain enigmatic due to the lack of a precise definition and the optimal methodologies to assess it. In this study, which was conducted from a neuroscientific standpoint, we defined consciousness of normal human participants as fluctuations in mental states that can be detected through assessments of brain activity and eyelid closure (EC).

In a report on consciousness (Havlik 2017), the “background states of consciousness” have been described as the states of being awake, asleep, dreaming, comatose, or in vegetative condition, which can appear both in healthy and pathological conditions. In the background states of consciousness, which range from low to high levels of vigilance, we focused on the distinction between arousal and drowsiness (i.e., light sleep), which is detectable as EC by using a near-infrared camera over the participant’s eye. The contrasting brain activity between these states measured by resting-state functional magnetic resonance imaging (rs-fMRI) may elucidate the neural correlates of consciousness.

The “general stream of consciousness” refers to a stream of mental states and ongoing subjective experiences (Havlik 2017). Since fluctuations in arousal are a major characteristic of consciousness, analysis of rs-fMRI time-series data is suitable for elucidating the nature of consciousness. For this purpose, we compared two time-series data obtained simultaneously—brain activity from the rs-fMRI and the degree of EC from a near-infrared camera—to predict subjective arousal based on the neural activity in a particular region of the brain.

The neural correlates of consciousness involve multiple regions in the brain, including the brain stem, thalamus, basal forebrain, and prefrontal cortices, and reciprocal transfer of information between these regions (Bogen 1995; Schiff 2008; Min 2010). Neurophysiological studies in monkeys and humans have shown that neuronal activity, represented by gamma band activity in the thalamus, is critical for conscious experiences (Boly et al. 2013). The thalamus plays a significant role in sensory integration as it receives multiple sensory information via cortical and subcortical pathways and sends processed information back to areas throughout the cortex (Schiff 2008; Min 2010; Jerath and Crawford 2014).

A meta-analysis of clinical studies assessing patients with disorders of consciousness revealed significantly reduced brain activity in the bilateral medial dorsal nuclei of the thalami in the patients when compared with that of the control participants (Hannawi et al. 2015). A systematic review of outcomes and electroencephalography (EEG) data in patients with disorders of consciousness (Kotchoubey and Pavlov 2018) has shown that the most predictable measure of recovery is sleep spindles, which is considered to originate from the activity in the thalamocortical loop (Fernandez and Luthi 2020).

Neuroimaging studies on sleep have shown a significant decrease in the subcortical and thalamic cerebral blood flow during nonrapid eye movement and an increased blood oxygen level–dependent (BOLD) activity in the thalamus, which was associated with the onset of sleep spindles and elevation of vigilance (Schiff 2008; Dang-Vu and Maquet 2010; Picchioni et al. 2013; Knaut et al. 2019). A study involving simultaneous recording of fMRI, EEG, and eye movement video showed that a brief episode of drowsiness was associated with a transient decrease in BOLD activity in the thalamus (Poudel et al. 2014). These results indicate that the thalamus plays a pivotal role in consciousness under physiological and pathological conditions.

Measurement of eye movement and pupil diameter by using a near-infrared camera in an magnetic resonance imaging (MRI) scanner is relatively easy to implement and requires minimal restriction of the participant’s head movement. Several neuroimaging studies have used video cameras to measure the degree of EC during fMRI scanning and have evaluated the percentage of time spent with eyes closed in a minute as a behavioral estimation of drowsiness (Poudel et al. 2012; Poudel et al. 2014; Wang et al. 2016; Poudel et al. 2018). In this study, we followed these eye-tracking methods to evaluate subjective drowsiness during fMRI scanning.

Another analytical approach involves predicting the subjective arousal level on the basis of fluctuations in the BOLD signal in a region of the brain at the individual level. In a study using macaque monkeys and simultaneous recordings of rs-fMRI and eyelid behavior, eye opening was associated with increased activity in the thalamus, while eye closure was associated with increased activity in the visual cortex (Chang et al. 2016). A brain template was created from a spatial pattern of activity that was associated with arousal/drowsiness and was applied to the time-series data of rs-fMRI scans of the participant. The time course of the estimated arousal level from the brain template successfully tracked a measure of behavioral arousal. Such a template-based approach was applied to human participants to predict the arousal level using rs-fMRI data (Falahpour, Chang, et al. 2018; Liu and Falahpour 2020).

The aim of this study was 2-fold: to delineate the region of the human brain that is dedicated to maintaining arousal and to predict arousal based on the neural activity in a brain template that was created using the outcome of the first question. For the first question, we hypothesized that the human thalamus is a key region of vigilance based on the aforementioned literature; therefore, we conducted a search restricted to the left and right thalami. Additionally, by applying a mask on the subnucleus of the human thalamus (Krauth et al. 2010), we could explore which subnuclei (i.e., mediodorsal nucleus [MD], pulvinar [PUL], and ventral anterior lateral nuclei group [VAL]) were the most relevant to arousal/drowsiness.

For the second question, we performed the receiver operating characteristic (ROC) analysis (Obuchowski 2003; Akobeng 2007) on two time-series data (i.e., template-based brain activity and arousal index data measured by eye-tracking) to compute the area under the curve (AUC) at the individual level. To avoid circular analysis of data, we applied the leave-one-out crossvalidation method to predict the arousal state from the brain activity. Our hypothesis was that in most participants, the ROC analysis could significantly predict the arousal level, and in a group analysis, the mean AUC could be significantly greater than the chance level.

## Materials and Methods

### Participants and Experimental Procedures

Twenty healthy individuals (all right-handed; males/females, 12/8; mean age, 23 years; standard deviation [SD], 1.5 years) participated in the experiment after providing written informed consent. The study was approved by the ethics committee of Nagoya University, School of Medicine, Nagoya, Japan. The participants’ brain was scanned at rest using a 3T MRI system (Siemens) at the Brain & Mind Research Center, Nagoya University. During the scan, they were instructed to stay awake and keep their eyes open and to avoid falling asleep as much as possible; however, they were allowed to blink when needed. The room was darkened by switching off all sources of light except for a small light within the scanner bore. A multiband echo planar image (EPI) acquisition (multiband factor = 3, time repetition [TR] = 1.0 s, time echo [TE] = 30 ms, flip angle = 55°, 64 × 64 matrix, 45 slices, voxel size = 3 × 3 × 3.5 mm) was conducted by covering the whole brain using a 32-channel head coil (Moeller et al. 2010; Xu et al. 2013). Simultaneously, the movement and pupil diameter of the participant’s left eye were monitored using an MRI-compatible eye-tracking device (Cambridge Research Systems) at a sampling rate of 60 Hz. The scan run was repeated either three (655 volumes for two participants) or four times (485 volumes for 18 participants). For the convenience of analysis, the 655-volume runs from the two participants were shortened to 485-volume runs by removing the last 170 volumes. Finally, high-resolution T1-weighted anatomical images (MPRAGE, TR = 2.5 s, TE = 4.38 ms, flip angle = 8°, 256 × 256 matrix, 192 slices, voxel size = 0.75 × 0.75 × 1 mm) were also acquired for each participant.

### Eye-Tracking Data Analysis

The eye-tracking system measured the movements of the eye in the horizontal and vertical directions and the pupil diameters at the maximum and minimum lengths at a sampling rate of 60 Hz. The pupil area was computed from the diameters, and the time-series data were smoothed by using nine time-point moving averages and were divided by the largest value in each scan to compute the normalized pupil area (nPA) (Chang et al. 2016). In the time-series data of nPA (range, 0–1), an EC period was defined as successive nPA values < 0.5. The EC periods were categorized as blinks if the duration was <2 s and drowsiness if the duration was ≥2 s. The 2-s threshold for distinguishing between blinks and drowsiness was determined empirically by visual inspection of all participants’ eye movement video data. The mean blink rate (per minute) and the proportion of instances of drowsiness during the scan time were computed and averaged across the 20 participants. The occurrence of blinks was treated as an event and drowsiness as a block in the statistical analysis of fMRI data.

Horizontal and vertical eye movement data were despiked, linearly interpolated, detrended, and smoothed using the nearest neighbor method. The principal component analysis (PCA) was applied to the data (Fransson et al. 2014) by using Origin Pro (ver. 2019; OriginLab, https://www.originlab.com/) to reduce dimensionality. We performed PCA of the horizontal and vertical components to capture the changes in eye position during the scan in a single regression vector. Subsequently, the vector was downsampled to 1 Hz to match the multiband fMRI sampling frequency (TR = 1 s). Next, the time-series were convolved with the canonical hemodynamic response function. Thus, a single time-series data that represented the overall degree of horizontal and vertical eye movements was used as a covariate in the fMRI statistical analysis.

### fMRI Data Analysis

The fMRI data for each participant were analyzed individually using SPM12 (https://www.fil.ion.ucl.ac.uk/spm). After discarding the first five scans to allow for stabilization of magnetization, the remaining scans were realigned, normalized to the Montreal Neurological Institute (MNI) template, and smoothed using an 8-mm Gaussian kernel. Slice-timing correction was not performed since the sampling rate was short (TR = 1 s). In the spatial normalization step, the mean EPI of each participant was coregistered onto a high-resolution anatomical image (MPRAGE), and a matrix obtained by a normalization process from MPRAGE to the MNI template was applied to all EPI data.

Statistical analyses were conducted using a general linear model. The SPM design matrix was created by including three regressors of interest: 1) blink condition as an event (onset-time only), 2) drowsiness condition as a block (both onset-time and duration), and 3) the degree of eye movement as a covariate, with 13 regressors of no interest (i.e., six movement parameters, its first temporal derivatives, and the global signal). Regression of the global signal from the time-series data has been a matter of debate in the analysis of rs-fMRI data. Several studies using human (Zhang, Huang, et al. 2020) and monkey (Scholvinck et al. 2010) subjects have indicated that the global signal has neuronal origin and correlates with the activity in the cortical regions. Therefore, to investigate whether the results of this study would be affected by global signal regression (GSR), we conducted the data analyses both with and without GSR.

Six contrast images were created for each participant: 1) positive correlation with the blink condition, 2) negative correlation with the blink condition, 3) positive correlation with the drowsiness condition, 4) negative correlation with the drowsiness condition, 5) positive correlation with eye movement, and 6) negative correlation with eye movement. In the following section, we refer to “negative correlation with the drowsiness condition” as “positive correlation with the arousal condition” High-pass-frequency filters (128 s) were applied to the time-series data to remove low-frequency noise. An autoregressive model was used to estimate the temporal autocorrelation.

In two participants, analysis of eye data revealed that there was no drowsiness condition during scanning; therefore, group analysis for the arousal condition was performed in the remaining 18 participants. The group analysis was conducted using the one-sample *t*-test (statistical threshold was set at *P* = 0.001, uncorrected, for the peak level, and *P* = 0.05, FWE-corrected for the cluster level) by applying a neuromorphometric atlas of the left and right thalami proper in SPM12. Hereafter, the cluster that survived the threshold within the thalamus is referred to as the thalamus mask.

**Figure 1:**
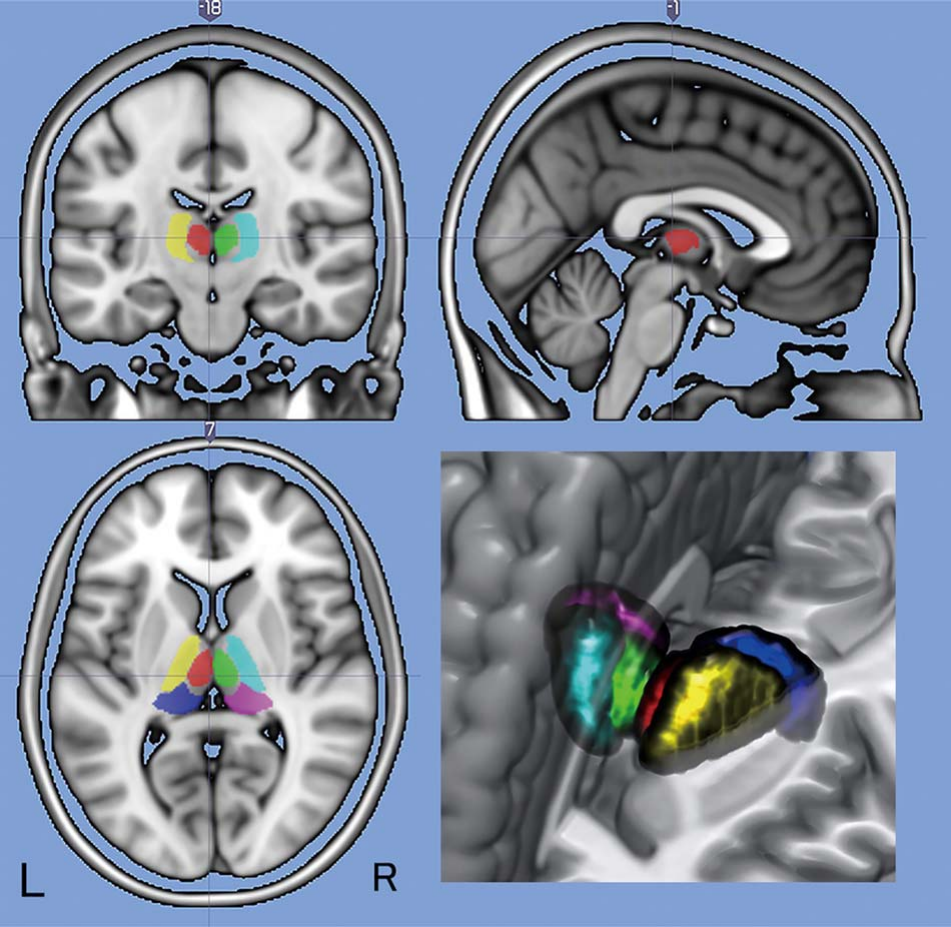
A 3D atlas of the human thalamus is superimposed on the MNI brain template of MRIcron. Some small subnuclei are merged into a large VOI for the MD (left, red; right, green), VAL (left, yellow; right, cyan), and PUL (left, blue; right, magenta) in the left and right hemispheres. Coronal (*y* = −18), sagittal (*x* = −1), and axial (*z* = 7) images and 3D rendering of the VOIs are shown in the figure.

Since several distinct thalamic subnuclei are known to play unique roles in consciousness, perception, and cognition (Bogen 1995; Schiff 2008; Min 2010; Saalmann and Kastner 2011), we applied a 3D atlas of the human thalamus (Krauth et al. 2010) to the results of the group analysis. The 3D atlas, which consists of 42 subnuclei in each hemisphere, was normalized to the MNI space with a voxel size of 1 × 1 × 1 mm. Considering the spatial resolution of the original EPI (voxel size: 3 × 3 × 3.5 mm), some small subnuclei were merged into a large volume of interest (VOI) for the MD, VAL, and PUL in the left and right hemispheres. The MD consists of magnocellular (MDmc) and parvocellular (MDpc) parts. The VAL consists of the ventral posterior nucleus, ventral lateral nucleus, ventral anterior nucleus, and ventral medial nucleus. The PUL includes the medial pulvinar, inferior pulvinar, lateral pulvinar, and anterior pulvinar. The volume (cubic mm) of each VOI was as follows: MD, left = 1190, right = 1197; VAL, left = 3202, right = 2380; and PUL, left = 2385, right = 3205. The spatial locations of the VOIs are shown on the canonical brain template of MRIcron (https://people.cas.sc.edu/rorden/mricron, Fig. 1).

The beta values associated with the arousal condition were extracted from each VOI and entered into a two-way analysis of variance (ANOVA), with the region and the hemisphere as factors (statistical threshold was set at *P* < 0.05, with correction of multiple comparisons using Fisher’s least-square method). The spatial overlap between the VOIs and the thalamus mask was explored using the MRIcron software. The overlapping region between the thalamus mask and the subnucleus VOI is shown in yellow, the nonoverlapping region of the thalamus mask is shown in green, and the nonoverlapping region of the subnucleus VOI is shown in red (see Fig. 6).

### Template-Based Prediction of Arousal

**Figure 2:**
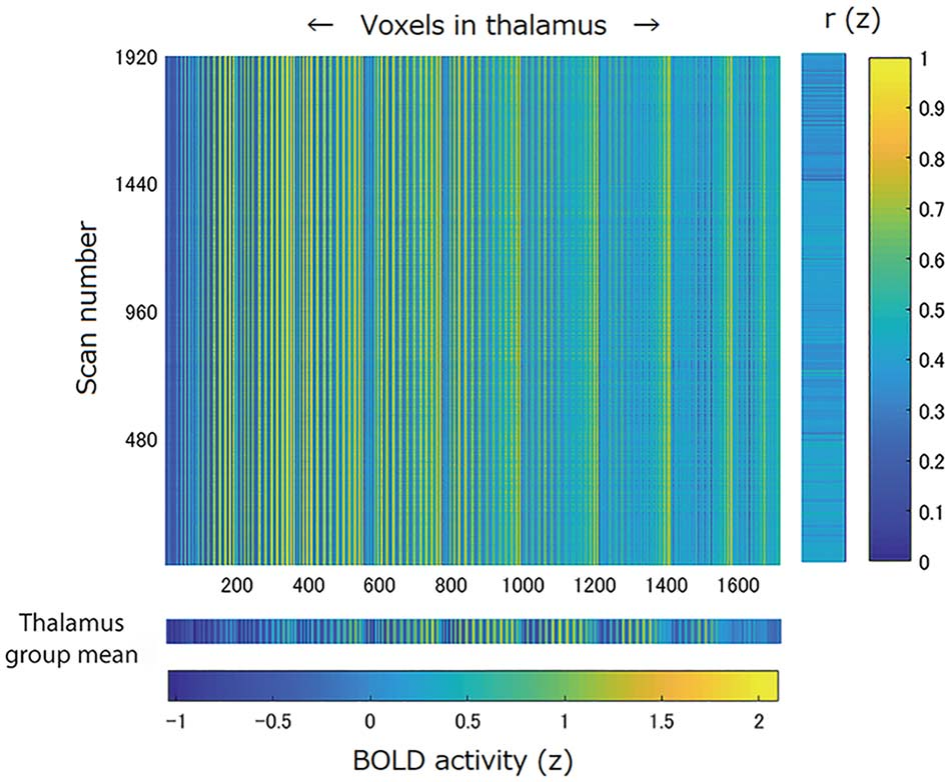
A large matrix with 1920 (scan number) rows and 1718 (voxels in the thalamus template) columns represents the data of a single participant with four scans. In an individual analysis, the time-series data were extracted from voxels in the thalamus template, *z*-transformed, and concatenated across the scans. A separate row in the bottom indicates z-transformed BOLD activity of the thalamus template (1718 voxels) averaged across the 17 participants in a group analysis (thalamus group mean). Pearson’s correlation coefficients between the thalamus group mean data and the individual thalamic activity data were computed from the first to the 1920th scan. The correlation coefficient was Fisher-transformed to create a single vector that represented the similarity between the template and an individual’s brain activity (a separate column on the right-hand side). The similarity vector [*r*(*z*)] was used in the ROC analysis.

Since we hypothesized that the time-series data of brain activity could predict fluctuations in consciousness between arousal and drowsiness, we analyzed the signal changes in the thalamus and the arousal defined from the eye-tracking data in each individual participant by using the ROC analysis (Obuchowski 2003; Akobeng 2007). The analysis was conducted using a template-based approach (Chang et al. 2016; Falahpour, Chang, et al. 2018; Liu and Falahpour 2020). Additionally, to avoid circular analysis of the data (i.e., data for prediction were also used to build the template), we applied the leave-one-out crossvalidation method. First, the template of the thalamus was created from the activated areas that were associated with the arousal condition in the group analysis of 17 participants at the same threshold as that with the group analysis of 18 participants. The beta values were extracted from every voxel in the template and were *z*-transformed by subtracting the mean and dividing by 1 SD. The *z*-transformed beta values within the thalamus template were averaged across the 17 participants and were reshaped as a single vector to create the group mean of the thalamus (Fig. 2, the second row from the bottom).

Second, in the individual analysis of a single participant who was excluded from the template-building process, the time-series data were extracted from every voxel within the thalamus template built from the remaining 17 participants and was *z*-transformed in each scan. The data were reshaped and concatenated across the scans to create a matrix (Fig. 2 shows an example of a single participant; a row and column indicate voxels in the thalamus and scan number, respectively). Subsequently, Pearson’s correlation coefficients between the group mean of the thalamus and the individual brain activity data were computed from the first to the last scans. The correlation coefficient was Fisher-transformed to create a single time-series data that represented the similarity between the template and individual brain activity (Fig. 2, indicated by *r*(*z*), the second column from the right). This procedure was independently repeated 18 times to compute the similarity regressor for each participant.

From the eye-tracking data of each participant, the start and end points of the drowsiness condition were determined by the time stamp. These time points were shifted by 4.5 s to account for the hemodynamic response delay of the BOLD signal. In the time-series of the scan, if an EPI volume was taken during the drowsiness block, it was labeled “0,” and it was otherwise labeled “1.” Thus, the binary data of a single vector indicating the participant’s arousal (drowsiness, 0; arousal, 1) was created for each scan, and the data were concatenated across scans. The binary arousal data were averaged across 78 scans for 20 participants to compute the time-course of the arousal level during the scan. A second-order polynomial function was fitted to the curve. This analysis was conducted to compare the probability of arousal during the scan between this study and a previous study (Tagliazucchi and Laufs 2014) by using simultaneous recordings of EEG and fMRI, which is the gold standard for measuring arousal.

The binary arousal data and similarity of brain activity based on the thalamus template were used for the ROC analysis (statistical threshold was set at *P* < 0.05). The AUC value, which indicates the degree of predictability (range, 0.5–1), was computed for each participant and was averaged. The mean AUC value was compared against a value of 0.5, which indicated that the prediction result was at a chance level, by using a one-sample *t*-test (statistical threshold was set at *P* < 0.05). The template-based prediction of arousal was conducted both with and without GSR.

## Results

### Arousal/Drowsiness Analysis by Pupil Diameter

**Figure 3:**
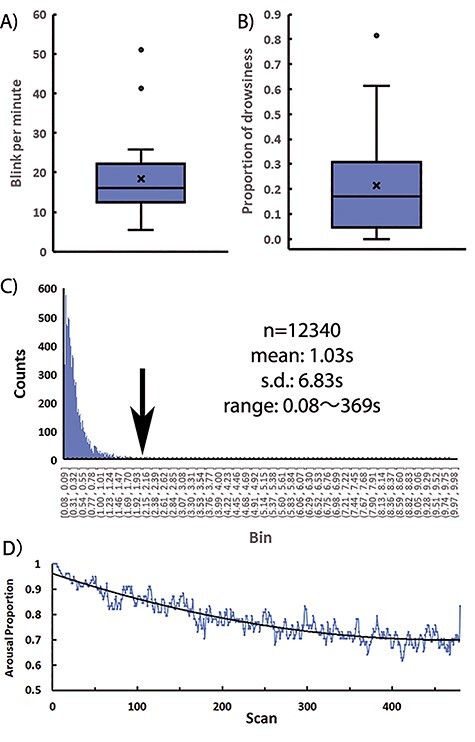
A box plot of the mean blink rate (time per minute, *A*) and the proportion of drowsiness during the scan time (*B*) for 20 participants. The box indicates the first and the third quartiles. The horizontal bar and the cross represent the median and mean, respectively. The whiskers indicate plus/minus 1.5× interquartile range. Dots indicate outliers. (*C*) The histogram of the EC period across 20 participants is shown. The horizontal and vertical axes indicate the time bin (0.01 s, from 0.08 to 9.98 s) and counts, respectively. The black arrow indicates the 2-s threshold that separates blinks and drowsiness. Total number, mean, SD, and range of the EC period are shown in the figure. Approximately, 7% of the EC periods are labeled as drowsiness. (*D*) The proportion of arousal periods during the scan averaged across 78 runs from 20 participants. The horizontal axis indicates the scan number, and the vertical axis indicates the proportion of aroused participants. A black curved line indicates a fitted second-order polynomial function (*Y* = 1E − 06*X*^2^ − 0.0011*X* + 0.9626, *R*^2^ = 0.87, *P* < 0.01).

The mean (SD) blink rate during the scan was 18.3/m (11.3/m) (Fig. 3*A*). The mean proportion of drowsiness during the scan was 0.21 (0.22), indicating that the participants remained awake during 79% of the scan time (Fig. 3*B*). There was a significant negative correlation between the mean blink rate and the proportion of drowsiness (*r* = −0.49, *P* < 0.05, *n* = 18). The histogram shows the count (vertical axis) and the duration (horizontal axis, time-binned in 0.01 s) of the EC periods summed across 20 participants (Fig. 3*C*, arrow indicates a duration of 2 s). The total number of EC periods was 12 340, with a mean duration of 1.03 s (6.83 s). The proportion of drowsiness conditions in all EC periods was 7.3%. The mean proportion of arousal during the scan across 20 participants gradually decreased from 1 to 0.7 (Fig. 3*D*). The data were fitted with a second-order polynomial function (*Y* = 1E-06*X*^2^ − 0.0011*X* + 0.9626, *R*^2^ = 0.87, *P* < 0.01).

### rs-fMRI Data Analysis Using GSR

**Figure 4:**
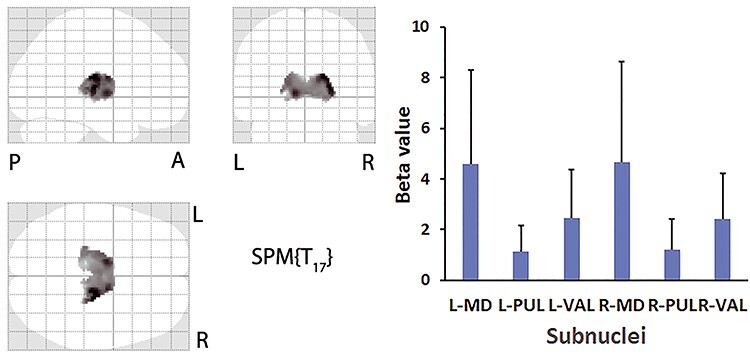
Left: A SPM glass-brain view of significant positive correlation with the arousal condition with GSR is shown (threshold at *P* = 0.001, uncorrected, for the peak-level, and *P* = 0.05, FWE-corrected, for the cluster level). The neuromorphometrics atlas of the left and right thalami proper in SPM12 is applied in the analysis. Right: The mean (column) and SD (bar) of the activation in each of the VOIs in the thalamic subnuclei are shown. L/R, left/right hemispheres.

**Figure 5:**
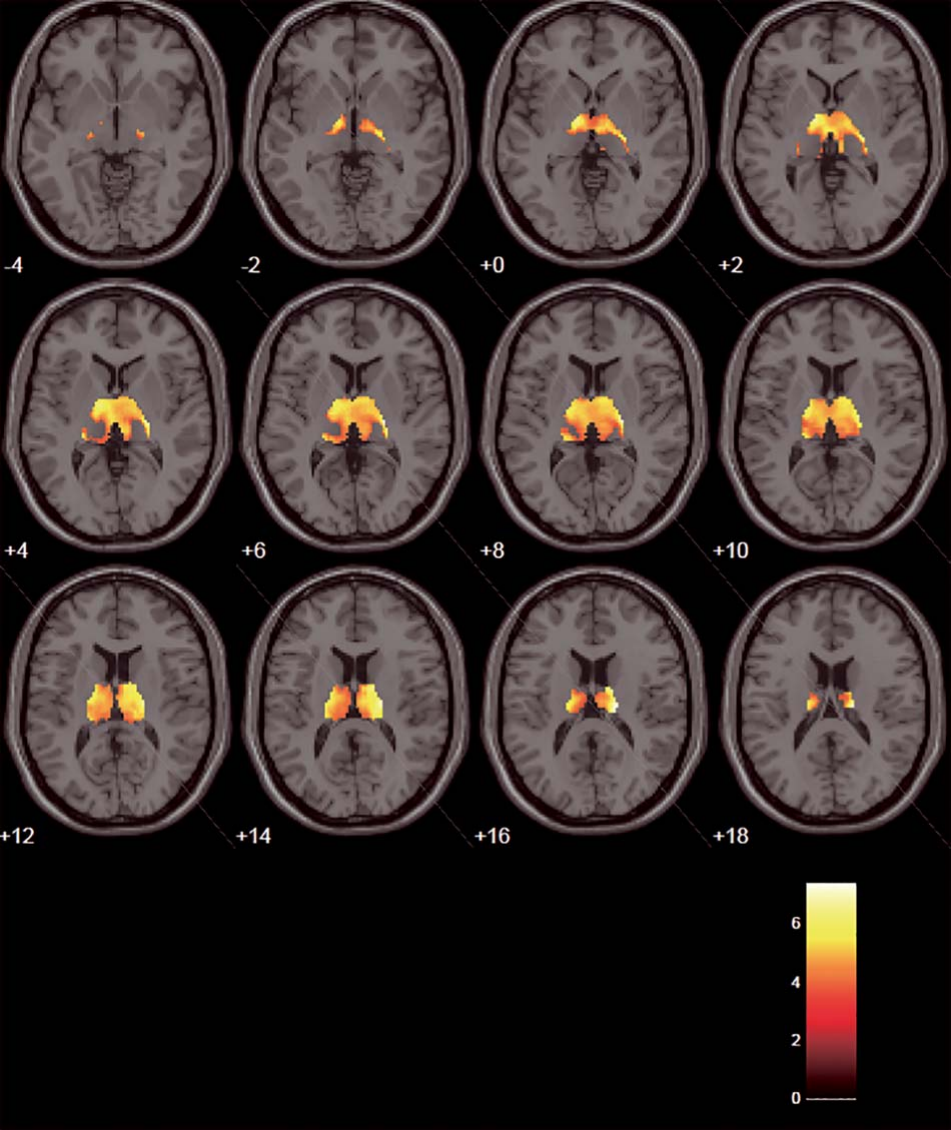
Significant positive correlation with the arousal condition in the thalamus is superimposed on the canonical brain of SPM12. The results are with GSR. The statistical threshold was set at *P* = 0.001, uncorrected, for the peak-level, and *P* = 0.05, FWE-corrected, for the cluster level. The left side on the figure shows the left side of the brain. Axial images are shown from *z* = −4 to *z* = 18 mm in 2-mm increments.

**Figure 6:**
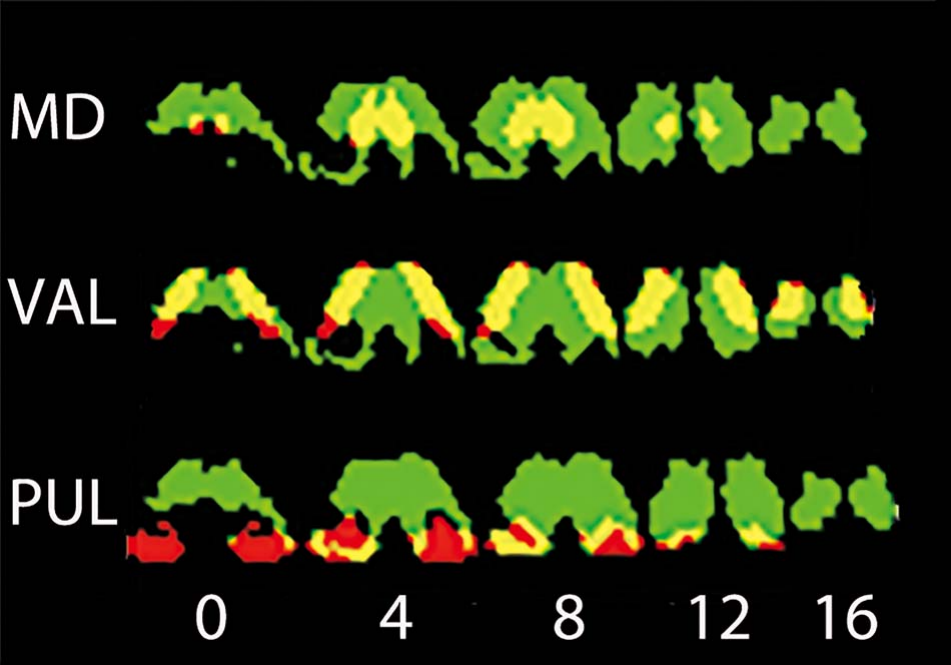
A regional overlap between the thalamus mask (significant positive correlation with the arousal condition at *P* = 0.001, uncorrected, for the peak-level, and *P* = 0.05, FWE-corrected, for the cluster level) and each of the VOIs (MD, VAL, and PUL) is shown. The thalamus mask is colored green, the VOIs red, and the overlap yellow. The MD shows the maximal overlap with the mask, while the PUL shows the least overlap, and the overlap of the VAL falls between those of the MD and PUL. Numbers in the bottom indicate levels at the *z*-axis (mm). The results are with GSR.

The analysis of fMRI data with GSR revealed a significantly positive correlation with the arousal condition in the thalami of the left and right hemispheres (*k* = 1797 voxels, Fig. 4, left panel, Fig. 5). The mean beta value extracted from the VOIs of the thalamic subnuclei is shown in Figure 4 (right panel). The results of the two-way ANOVA revealed a significant main effect of region (*F*[2, 102] = 16.9, *P* < 0.001), though no significant main effect of hemisphere (*F*[1, 102] = 0.008, *P* = 0.93) or their interaction (*F*[2, 102] = 0.005, *P* = 0.99). A post hoc analysis with correction of multiple comparisons revealed that the mean beta value in the MD was greater than that in the VAL and PUL (both *P* < 0.001), and the mean beta value in the VAL was greater than that in the PUL (*P* < 0.05). To explore the spatial overlap between each subnucleus and thalamus mask, the VOIs were overlaid (yellow in Fig. 6). The MD showed maximal overlap with the mask, the PUL showed the least overlap, and the VAL showed an overlap between those of the MD and VAL (Fig. 6). There was no significant cluster in the thalamus that showed positive correlation with drowsiness (*P* = 0.001, uncorrected, for the peak level).

### rs-fMRI Data Analysis without GSR

The analysis of fMRI data without GSR revealed a significantly positive correlation with the arousal condition in the thalami of the left and right hemispheres (*k* = 748 voxels, Supplementary Fig. S1: left panel, Supplementary Fig. S2). The mean beta value extracted from the VOIs of the thalamic subnuclei is shown in Supplementary Figure S1 (right panel). The results of the two-way ANOVA revealed a significant main effect of region (*F*[2, 102] = 14.7, *P* < 0.001); however, there was no significant main effect of the hemisphere (*F*[1, 102] = 0.006, *P* = 0.94) or their interaction (*F*[2, 102] = 0.003, *P* = 0.99). A post hoc analysis with correction of multiple comparisons revealed that the mean beta value in the MD was greater than that in the VAL (*P* < 0.005) and PUL (*P* < 0.001), and the mean beta value in the VAL was greater than that in the PUL (*P* < 0.05). The MD showed small overlap with the mask, and the PUL showed a negligible overlap; the VAL showed an overlap between those of the MD and VAL (Supplementary Fig. S3). There was no significant cluster in the thalamus that showed positive correlation with drowsiness (*P* = 0.001, uncorrected, for the peak level).

### Template-Based Prediction of Arousal with GSR

**Figure 7:**
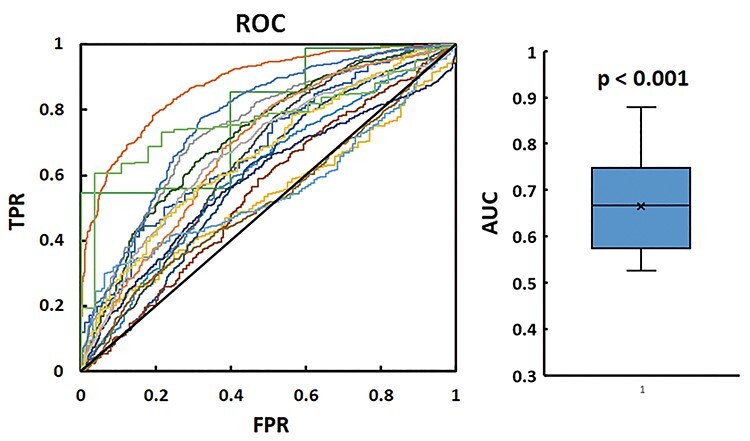
Left: The results of the ROC analysis with GSR for each participant (*n* = 18) are shown. The horizontal and vertical axes indicate the false-positive rate (FPR) and true-positive rate (TPR), respectively. Each colored and curved line represents the result for each participant. In 16 of the 18 participants, the AUC was significantly (*P* < 0.05) greater than the chance level (0.5). A diagonal black line indicates a chance level of prediction. Right: A box plot of the AUC value across the 18 participants is shown. The mean AUC was significantly (*P* < 0.001) greater than the chance level (0.5).

The results of the ROC analysis with GSR showed that the degree of similarity with the thalamus template significantly predicted arousal in 16 of 18 participants (*P* < 0.05, Fig. 7, left panel). The mean AUC value was 0.66 (0.10), which was significantly greater than 0.5 (*P* < 0.001, Fig. 7, right panel). The mean cutoff point (i.e., nearest point to the left top corner of the ROC plot) was 0.32 (0.18).

### Template-Based Prediction of Arousal without GSR

The results of the ROC analysis without GSR showed that the degree of similarity with the thalamus template significantly predicted arousal in 15 of 18 participants (*P* < 0.05, Supplementary Fig. S4, left panel). The mean AUC value was 0.65 (0.11), which was significantly greater than 0.5 (*P* < 0.001, Supplementary Fig. S4, right panel). The mean cutoff point was 0.33 (0.21). There was no significant difference in the mean AUC value between the analyses with and without GSR (paired *t*-test, *P* = 0.49, Supplementary Fig. S5). Thus, our results demonstrated that the template-based approach successfully predicted arousal in most participants, which also applied to the group analyses both with and without GSR.

## Discussion

This study showed that the participants’ arousal state, in comparison with the drowsy state, was associated with activity in the bilateral thalami. The results indicated, in accordance with the findings of previous studies, a pivotal role of the thalamus in mediating the arousal state in healthy human participants. In addition, the findings demonstrated that among the major subnuclei of the thalamus, the MD is the most relevant to the arousal state when compared with the PUL and the VAL. These findings were revealed for the first time by using a detailed 3D atlas of the thalamic subnuclei. Finally, the ROC analysis with leave-one-out crossvalidation using a template-based approach and eyelid behavior successfully predicted the participant’s arousal at both the individual and group levels. These results were confirmed by analyses performed both with and without GSR.

The significant predictability in this study might have been due to the fact that in the fMRI statistical analysis, we included both blinking and drowsiness, both of which were measured using an eye-tracking system as regressors. This approach may clearly separate the activity in each condition, which significantly differs in terms of temporal characteristics. In the following section, we discuss the results of eye-tracking, the significance of the major thalamic subnuclei in consciousness, the findings of the ROC analysis using a template-based approach, the effects of GSR, and the implications in clinical neuroscience.

### Eyelid Behavior

The rate of spontaneous eye blinks in healthy human participants has been investigated in relation to development, aging, cognitive performance, and personality traits, as well as neurological and psychiatric disorders, such as Parkinson’s disease, schizophrenia, and autism. Although the mean eye blink rate (times per minute) depends on age, sex, and other factors, it is ~20 times per minute (Jongkees and Colzato 2016). In a review article on spontaneous eye blink activity, a scatterplot of data from the literature on normal healthy participants indicated that in adults aged 20 years, the estimated rate is ~17 times per minute (Cruz et al. 2011). The rate observed in this study, 18.3 times per minute, was within the ranges reported in previous studies, indicating that the distinction between blinking and drowsiness was appropriate.

The mean proportion of arousal during the scan time was 0.79, and the proportion decreased nonlinearly from 1 at the start time to ~0.7 after 480 s. In a study using simultaneous recordings of EEG and fMRI, the proportion of arousal confirmed by EEG was 60% during the scan time (Tagliazucchi and Laufs 2014), which was slightly lower than that observed in this study. However, Tagliazucchi et al. (Tagliazucchi and Laufs 2014) used a long scan time (52 min), whereas we used a relatively short scan time (8 min) in this study. The long scan time likely induced drowsiness in the participants. The probability plot shows that ~70% of the participants were awake at 10 min (Tagliazucchi and Laufs 2014); therefore, the results of our study were similar to those obtained in the EEG–fMRI experiment. In addition, the significant negative correlation between the blink rate and proportion of drowsiness during the scan suggests that the frequency of blinks was related to wakefulness.

### Roles of Thalamic Subnuclei in Consciousness

#### Mediodorsal Nucleus

The anatomical subregions of MD consist of MDmc and MDpc parts, both of which are richly connected to the prefrontal cortex (PFC). The MDmc–PFC connections are almost reciprocal between the MDmc and the orbitofrontal cortex (OFC) and ventromedial PFC (vmPFC); however, there is nonreciprocal input from the ventrolateral PFC (vlPFC) and medial PFC (mPFC). The MDmc has projections to the limbic system, including the amygdala and entorhinal and perirhinal cortices in the medial temporal lobe. The MDpc is connected to the dorsolateral PFC (dlPFC), OFC, vlPFC, and dorsal anterior cingulate cortex; however, there are no projections to the limbic system. These patterns demonstrate that the MDmc is mainly connected with the ventral, medial, and orbital PFC, as well as the limbic areas, while the MDpc is connected with the dlPFC and vlPFC, though not with the limbic system (Mitchell and Chakraborty 2013; Ferguson and Gao 2014; Mitchell 2015; Bordes et al. 2020; Georgescu et al. 2020).

Such connectivity patterns indicate that the MD may engage in the modulation of various cognitive functions operated in each of the PFC areas where the MD sends or receives projections. Studies on monkeys have demonstrated that artificial lesions in the MD cause impairments in working memory, decision-making, executive function, associative learning, and resistance to interference (Mitchell and Chakraborty 2013; Ferguson and Gao 2014; Mitchell 2015; Bordes et al. 2020; Georgescu et al. 2020). In human studies, cognitive impairments reported after MD damage are related to deficits in working memory function and problems with complex associative memory, which are similar to the problems reported after frontal lobe damage (Mitchell 2015). Although other PFC functions, such as executive function, attention control, prospective memory, and motivation, are often reported in the acute phase of focal MD lesions, the symptoms most likely disappear in the chronic phase (Pergola et al. 2018).

In a study using simultaneous recordings of fMRI and EEG, BOLD activity in the medial dorsal nucleus of thalamus was observed to correlate significantly and positively with the EEG alpha power measured from the electrodes in the occipital lobe (Liu et al. 2012). These results corresponded to those in our study in that as the neural activity in the thalamic subnucleus increased, the participant’s level of vigilance increased. The authors noted that although the thalamus has been proposed to play an active role in generating alpha activity in the occipital lobe, the MD nucleus is more likely connected with other cortical and subcortical arousal systems in the brain. Therefore, significant association between the MD nucleus of the thalamus and vigilance would be caused by an elevation in cortical arousal in general and not solely by activation of the occipital lobe.

What is the role of the MD in keeping us conscious and awake? As the participants in this study were scanned at the resting state and did not engage in any cognitive tasks involving working memory, decision-making, executive function, etc., it is difficult to consider these cognitive demands when the activity of the MD increases during arousal. A possible explanation is that the MD is not a primary site of higher-order brain function but engages in subserving, maintaining, and extending cognitive operations in single or multiple PFC regions. A wide variety of projections connecting the PFC and MD may facilitate such cognitive processes in humans and animals; therefore, the role of the MD could not be determined definitively. The MD may actively enhance PFC excitability both spatially and temporally and allow continuous mental operations in a more elaborate manner (Pergola et al. 2018). Such MD functions would be activated before the commencement of an actual cognitive task to ensure readiness for effective operation of outgoing and incoming information to and from the PFC.

The cellular and neurophysiological underpinnings of the MD functions for arousal are still elusive; however, thalamic neurons are known to have two firing modes, namely, tonic and burst modes, and are capable of switching between the modes depending on the situation (Jones 2009). When the brain is in an awake and attentive state, cortically projecting thalamic neurons are capable of relaying information from sensory organs with high spatial and temporal resolutions, and prominent tonic activity emerges subsequently. By contrast, when the brain is in a drowsy and inattentive state, high-frequency bursts of action potential are discharged. The existence of two different modes of action potential and the capacity to switch between them may be the neurophysiological basis of the activity in the MD during the arousal state; however, further investigation is needed to clarify this model.

#### Pulvinar Nucleus

The ventral part of the PUL is connected to the occipital and inferior temporal cortices and superior colliculus, while the dorsal part is connected to the frontal, parietal, and superior temporal cortices. These connectivity patterns indicate that the ventral part could be dedicated to the feedforward and feedback processing of visual information (Saalmann and Kastner 2009, 2011) and that the dorsal part could be dedicated to hand–eye coordination in visually guided movements (Wilke et al. 2010). The PUL is considered a subcortical component of the attention network of the brain because it shows reciprocal connectivity with the prefrontal and superior parietal cortices. Several experiments in monkeys and humans have shown that PUL activity increases or decreases in response to attention modulation during behavioral tasks (Saalmann and Kastner 2009).

In this study, the mean beta value and the overlapping areas with the thalamus mask were smaller in the PUL than in the MD and the VAL, suggesting a minor role of the PUL in arousal in human participants. However, the overlapping regions, as illustrated in yellow in Figure 6, indicate that the activated areas were located mostly in the dorsal part and not in the ventral part of the PUL (*z* ≥ 4 mm in Fig. 6). Since the dorsal PUL predominantly connects with the prefrontal (e.g., frontal eye field and dorsolateral frontal cortices) and parietal (e.g., lateral intraparietal area) cortices (Saalmann and Kastner 2009; Wilke et al. 2010), it is very likely that the PUL supports functions of the dorsal attention and salience networks (Raichle 2015). Therefore, the PUL may contribute to arousal by subserving the prefronto-parieto-thalamic loop.

However, in a study using simultaneous recordings of fMRI and EEG, BOLD activity in the PUL correlated negatively with the EEG alpha power measured in the occipital electrodes (Liu et al. 2012), indicating an opposed finding in this study. The discrepancy in the results between the studies might be associated with the experimental conditions in which the participants were scanned during resting or viewing checkerboard patterns (Liu 2012) and during spontaneous fluctuation of arousal in a darkened room (in this study). The amount of visual input that stimulates the occipital cortical areas might have been greater in the study by Liu et al. (2012) when compared with that in this study. In terms of the visual input to the participant, it should be noted that the effects of arousal level and those of shutting visual input out from the eyes could not be segregated in this study.

#### Ventral Anterior Lateral Nuclei Group

This group of nuclei is connected to the basal ganglia, cerebellum, motor-premotor cortex, and somatosensory cortex and exerts functions of simple and complex behaviors and movements as well as sensations for the body, head, neck, and extremities (Bordes et al. 2020). Some of the nuclei in this group are focused on neuromodulation to ameliorate somatosensory and movement disorders, such as chronic pain, Parkinson’s disease, essential tremor, and Tourette syndrome (Schmid et al. 2016; Lee et al. 2019). In these patients, thalamic stimulation during surgery for the treatment of disorders produced sensations in touch, pressure, pain, vibration, and movements, and somatosensory stimuli in the participant’s body and evoked neural activity in the thalamus (Schmid et al. 2016). Therefore, it is reasonable that these nuclei are activated during arousal more than during drowsiness because the arousal of the brain makes it ready for processing somatosensory and movement information. In a study using simultaneous recordings of fMRI and EEG, BOLD activity in the anterior nucleus correlated positively with the EEG alpha power (Liu et al. 2012).

### Prediction of Arousal Using a Template-Based Approach

In an original report of a template-based approach to predict subjective arousal from rs-fMRI data (Chang et al. 2016), four monkeys sitting in a near-completely darkened room were scanned for ~30 min with no constraint regarding eye behavior. In a second study in which the same method was applied for the first time to human participants (Falahpour, Chang, et al. 2018), 10 participants were scanned twice for 5 min each with instructions for eye opening or closing. In our study, 20 human participants were scanned three or four times for 8 min each in a darkened room with clear instructions for eye opening. Therefore, the advantages of our study were the strong statistical power, which was almost double that of previous human fMRI studies (Falahpour, Chang, et al. 2018), and a total scan time that was as long as that in the study on monkeys (Chang et al. 2016).

In pioneering studies (Chang et al. 2016; Falahpour, Chang, et al. 2018), a correlational approach was used to predict the participant’s arousal from the rs-fMRI data. We conducted the ROC analysis, which is suitable for the evaluation of diagnostic tests with dichotomous outcomes by using eye behavior as the gold standard and revealed that, in most participants, the prediction result was significant. Binary outcomes of arousal and drowsiness, as defined by eye-tracking data, could be appropriate for such analyses. In line with the previous studies, to avoid inflation of the prediction accuracy, we applied the leave-one-out crossvalidation method for prediction of arousal by excluding one participant in the study set from being tested.

### Effect of GSR on the Association between Arousal and the Thalamus

In this study, the data analyses were conducted both with and without GSR. In the whole group analysis, the volumes of positive correlation in the thalamus during arousal with and without GSR were 1797 and 748 voxels, respectively. The mean beta values within the thalamic subregions were greater with GSR than those without GSR. In a study using simultaneous recordings of fMRI and EEG during eyes-closed sessions, a positive correlation between the participant’s vigilance and BOLD activity in the thalamus did not differ between the conditions with and without GSR (Falahpour, Nalci, et al. 2018). The authors explained that, due to a weak correlation between the thalamic BOLD signal and global signal in the whole brain, the effect of GSR on the activity pattern in the thalamus was minimum.

By contrast, in this study, activated volumes of the thalamus increased by 2.4-fold after the application of GSR when compared with the volume before GSR. The discrepancy in the effects of GSR between our study and the previous study (Falahpour, Nalci, et al. 2018) might have been due to the difference in the methodology to measure arousal (eye-tracking vs. EEG), eye conditions (open vs. closed), and data analysis (general linear model vs. correlational approach). By contrast, the GSR effect was small in the ROC analysis that used brain activity and arousal data since the mean AUC value did not differ significantly between the conditions with and without GSR (Supplementary Fig. S5). Therefore, prediction of arousal using the leave-one-out crossvalidation method might be insensitive to the effect of GSR.

### Implications for Clinical Neuroscience

Tracking arousal of human participants during rs-fMRI measurements is a topic of major research interest because neural networks at rest have been considered as biomarkers for neuropsychiatric diseases (Sha et al. 2018; Parkes et al. 2020). It remains unclear whether the aberrant networks observed in patients when compared with those in controls could have pathophysiological implications or simply be derived from the differences in arousal levels between the groups. In this sense, our study may provide further insights for investigating the differences in neuronal networks between healthy controls and diseased populations. As shown in our results, although the study was restricted to a healthy young population, the participants were in a drowsy state for ~21% of the scan time. As such, drowsiness could alter the functional connectivity within the brain, and the proportion of drowsiness may differ between the patients and controls. Measurement and evaluation of the vigilance level during scanning should be necessary for future studies.

Second, numerous studies have emphasized the abnormal size, function, and connectivity of either the thalamus or thalamocortical circuits in neuropsychiatric disorders, such as schizophrenia, depression, autism, and Alzheimer’s disease (Parnaudeau et al. 2018). In these disorders, deficits in higher-order cognition involving attention, working memory, and executive function are prevalent, and the causes of these impairments appear to be malfunctioning of the thalamus and thalamocortical circuits (Dorph-Petersen and Lewis 2017; Parnaudeau et al. 2018). Among the thalamic subnuclei, the MD is the most likely candidate for the neurophysiological basis of schizophrenia because it has strong connectivity with the PFC and limbic system, including the amygdala. If the levels of vigilance are controlled by the MD, investigations of structural and functional alterations of the MD and its connectivity patterns could reveal the pathogenesis of schizophrenia (Zhang, Quinones, et al. 2020).

## Conclusion

Simultaneous recordings of brain activity and eye behavior in healthy human participants revealed that the thalami in the left and right hemispheres were significantly activated during the arousal condition in comparison with that during the drowsiness condition. Among the major thalamic subnuclei, the MD was most significantly involved in arousal. These results may be attributable to the strong connectivity of this nucleus with the prefrontal and limbic areas, where higher-order cognitive and emotive functions are operated. The MD may indirectly exert itself to subserve, mediate, and maintain these functions before the commencement of a more demanding process in the brain. Finally, compared with a correlational analysis, an ROC analysis using a template-based approach and eyelid behavior successfully predicted subjective arousal from brain activity in a more elaborate manner. These findings suggest that the connectivity and network metrics obtained from rs-fMRI data are confounded by the levels of subjective arousal; therefore, these factors should be considered when rs-fMRI data are used as a biomarker of neuropsychiatric disorders.

## Notes

Multiband EPI software was provided by the University of Minnesota Center for Magnetic Resonance Research. A 3D atlas of the human brain was provided by Prof. Axel Krauth, Computer Vision Laboratory, ETH Zurich, Switzerland. We thank Prof. Minoru Hoshiyama for his technical help in the experiment. *Conflict of Interest:* None declared.

## Funding

JSPS KAKENHI (Grant Number 18K07347).

## Supplementary Material

Supplementary_materials_tgab055
