# Supplementary material for: Fluctuations in Arousal Correlate with Neural Activity in the Human Thalamus
Source: Cereb Cortex Commun. 2021 Sep 1;2(3):tgab055. doi: 10.1093/texcom/tgab055 (PMC8455340; doi:10.1093/texcom/tgab055)
Supplement: Supplementary_materials_tgab055 [file supplementary_materials_tgab055.docx]

Supplementary Figure 1


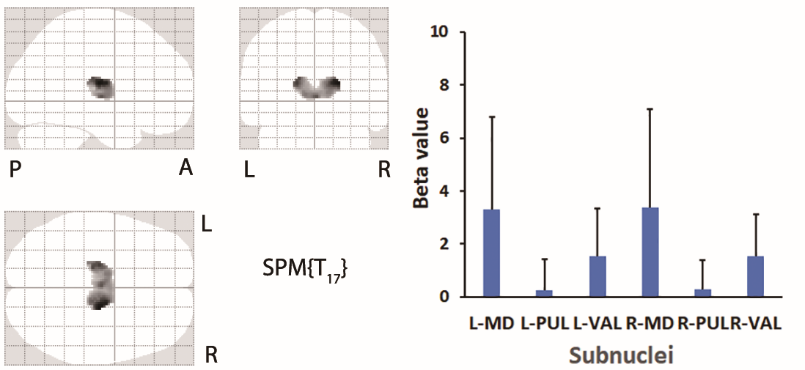


Left: An SPM glass-brain view of significant positive correlation with the arousal condition without GSR is shown (threshold at p = 0.001, uncorrected, for the peak-level, and p = 0.05, FWE-corrected, for the cluster level). The neuromorphometrics atlas of the left and right thalamus proper in SPM12 is applied in the analysis. Right: The mean (column) and s.d. (bar) of the activation in each of the VOIs in the thalamic subnuclei are shown. L/R, left/right hemisphere; MD, mediodorsal nucleus; VAL, ventral anterior lateral nuclei group; PUL, pulvinar.

Supplementary Figure 2


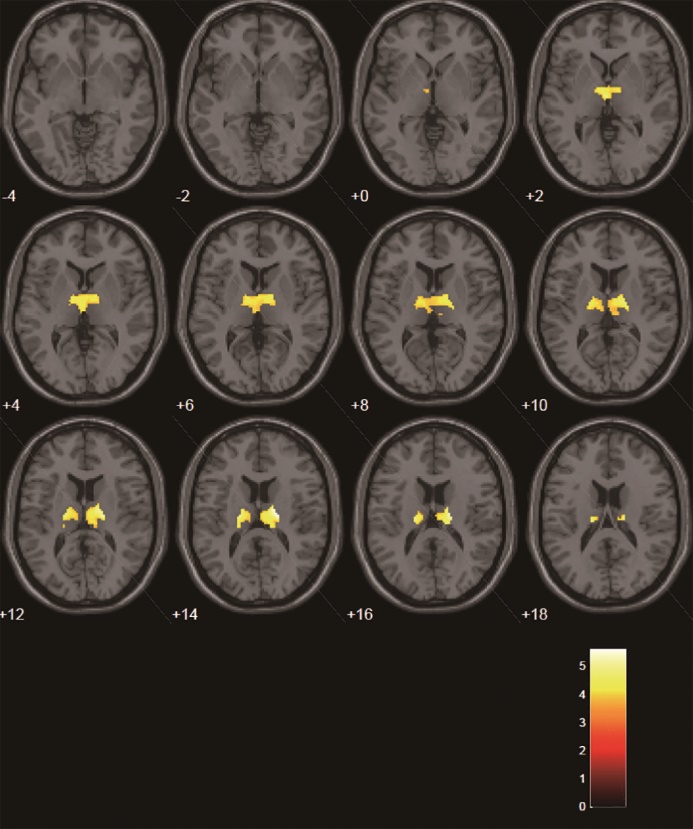


Significant positive correlation with the arousal condition in the thalamus is superimposed on the canonical brain of SPM12. The results are without GSR. The statistical threshold was set at p = 0.001, uncorrected, for the peak-level, and p = 0.05, FWE-corrected, for the cluster level. The left side on the figure shows the left side of the brain. Axial images are shown from z = -4 mm to z = 18 mm in 2-mm increments.

Supplementary Figure 3


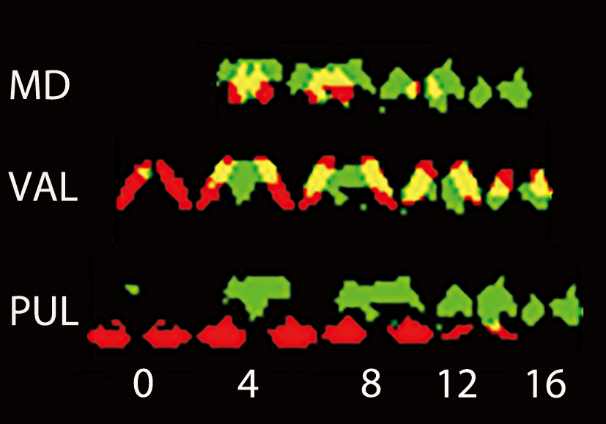


A regional overlap between the thalamus mask (significant positive correlation with the arousal condition at p = 0.001, uncorrected, for the peak-level, and p = 0.05, FWE-corrected, for the cluster level) and each of the VOIs (MD, VAL, and PUL) is shown. The thalamus mask is colored green, the VOIs red, and the overlap yellow. Numbers in the bottom indicate levels at the z-axis (mm). The results are without GSR.

Supplementary Figure 4


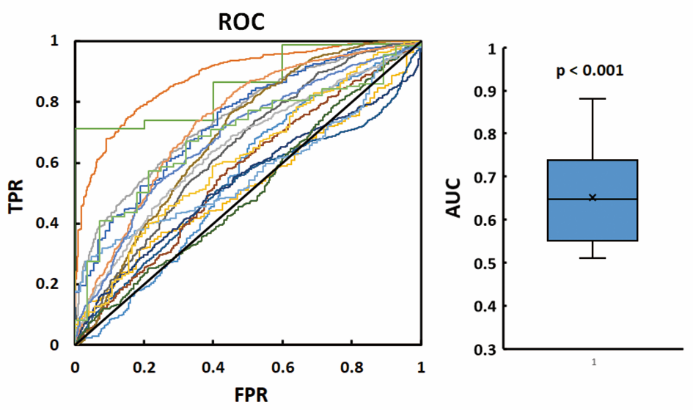


Left: The results of ROC analysis without GSR for each participant (n = 18) are shown. The horizontal and vertical axes indicate the false-positive rate (FPR) and true-positive rate (TPR), respectively. Each colored and curved line represents the result for each participant. In fifteen of the 18 participants, the AUC was significantly (p < 0.05) greater than the chance level (0.5). A diagonal black line indicates a chance level of prediction. Right: A box plot of the AUC value across the 18 participants is shown. The mean AUC was significantly (p < 0.001) greater than the chance level (0.5).

Supplementary Figure 5


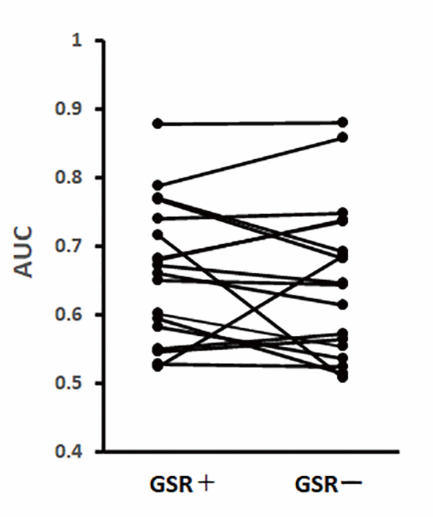


The plot shows the AUC value of 18 participants with (GSR+) and without (GSR‒) GSR. The bar connects the same participants with and without GSR. There was no significant difference in the mean AUC value (p = 0.49, paired t-test) between the conditions with and without GSR.

Supplementary Figure 6


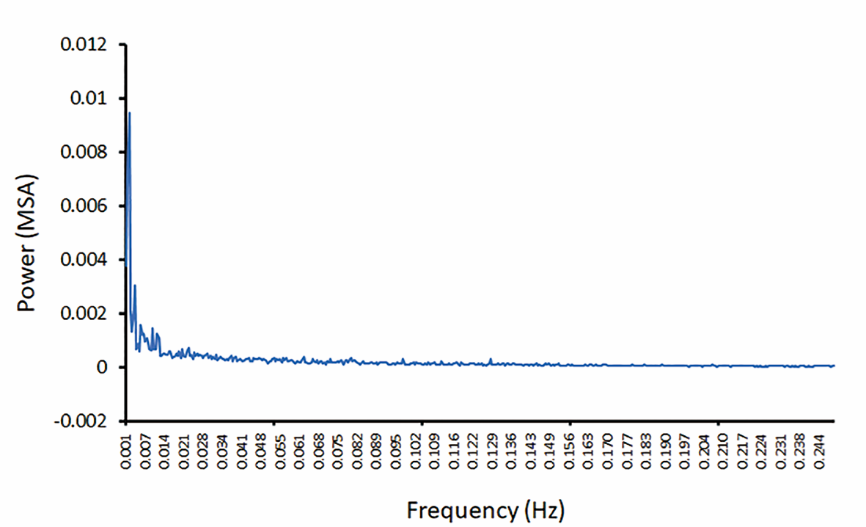


The mean power spectrum of arousal regressor.

Supplementary information

*1. Collinearity between the regressors*

It appears that there was some degree of correlation between the arousal and eye movement regressors. A high degree of correlation between the regressors leads to problems in estimation of parameters and the associated variances and affects the p-values. For linear regression, the variance inflation factor [VIF = 1/(1-r^2^)] is generally used as a measure to assess the degree of multicollinearity. A VIF value >10 generally indicates the use of a remedy to reduce multicollinearity (Bayman and Dexter, Multicollinearity in Logistic Regression Models, Anesthesia & Analgesia, (2021), 133, 2, 362-365). In our dataset, the VIF for the arousal and eye movement regressors ranged from 1.00−1.26. Therefore, it is unlikely that there was a high degree of collinearity between these regressors.

*2. The frequency analysis of arousal regressor*

The plot in Supplementary Figure 6 shows the mean power spectrum of arousal regressor for 17 participants who underwent four experimental runs (1920 time-points). The horizontal and vertical bars represent frequency (Hz) and power (MSA), respectively. The power was the strongest at low frequencies and subsequently declined towards high frequencies. The FFT was conducted using Origin Pro (ver. 2019; OriginLab, https://www.originlab.com/).
